# Profiles and spatial distributions of heavy metals, microbial communities, and metal resistance genes in sediments from an urban river

**DOI:** 10.3389/fmicb.2023.1188681

**Published:** 2023-06-29

**Authors:** Lingfang Fu, Yang Yu, Fei Yu, Jieer Xiao, Huaiyang Fang, Weijie Li, Zhijie Xie, Feng Zhang, Shu Lin

**Affiliations:** State Environmental Protection Key Laboratory of Water Environmental Simulation and Pollution Control, The Key Laboratory of Water and Air Pollution Control of Guangdong Province, South China Institute of Environmental Sciences, Ministry of Ecology and Environment, Guangzhou, Guangdong, China

**Keywords:** metal resistance genes, heavy metal, chemical fractions, urban river, bacterial community

## Abstract

The occurrence and propagation of resistance genes due to exposure to heavy metals (HMs) in rivers is an emerging environmental issue. Little is known about resistance genes in microbial communities in river sediments with low HM concentrations. The profiles and spatial distributions of HMs, the microbial community, and metal resistance genes (MRGs) were analyzed in sediment samples from the Zhilong River basin in Yangjiang city, near the Pearl River Delta. Concentrations of copper (Cu), cadmium (Cd), lead (Pb), chromium (Cr), and nickel (Ni) were relatively low compared with those in other urban river sediments in China. HM chemical composition and fractions and the structure of the microbial community varied along the main channel, but the composition and abundance of MRGs were relatively homogeneous. Variations in HMs and microbial communities in mid- to upstream areas were related to the presence of tributaries, whose inputs were one of the major factors affecting HM chemical fractions and genera structure in mainstream sediments. There were no significant correlations (*p* < 0.05) between HM concentrations, bacterial communities, and the MRG profiles; thus, HM concentrations were not the main factor affecting MRGs in sediments. These results contribute to understanding the propagation of MRGs in urban rivers in developing cities.

## 1. Introduction

Heavy metal (HM) contamination in aquatic environments is a serious concern (Huang et al., 2019; Xu et al., 2020). Metals in water easily adsorb to suspended particles and thus accumulate and become concentrated in sediments (Xiao et al., 2021). Metals in sediments naturally occur due to erosion of soil and rocks, supplemented by anthropogenic sources such as industrial effluent, mining activities, agricultural runoff, and leaching from urban waste materials (Omwene et al., 2018; Guo et al., 2019; Song et al., 2019).

Significant spatial variations in HMs can be observed within the river basin among upstream, midstream, and downstream areas (Xiao et al., 2021). The downtown areas of the city are more strongly affected by urbanization and anthropogenic activities. Fewer variations and heterogeneity in HM concentrations can be seen in more tourist attraction areas of the city with fewer sources of contamination. Aside from these land uses and sources, hydraulic factors such as tidal action mainly affect the downstream area.

Metal resistance genes (MRGs) occur naturally in organisms to reduce the impacts of HMs and are found extensively in aquatic environments, particularly in heavily polluted regions, such as areas contaminated by mine tailings and e-waste (Jie et al., 2016; Liu et al., 2018; Jin et al., 2022). Globally, multi-metal, mercury, and copper (Cu) resistance genes have been found to have higher relative abundances in lakes (Guo et al., 2019). Evaluating the co-occurrence and distributions of HMs and MRGs in various types of river sediments is important for understanding contaminant migration and transformation, but there is a relative lack of such research in river sediments.

Throughout history, urban rivers have facilitated socioeconomic development by providing valuable water resources. Yangjiang city has been relatively slow to industrialize and urbanize (Li et al., 2022), but will become more fully developed as an important regional center in the Pearl River Delta. As the mainstream river in this region, Zhilong River was chosen for this study. The Zhilong River flows from a valley in the upper watershed through the county to the sea, with significant spatial variations in land use and hydrology. The main aims of this study were to: 1) analyze the occurrence and distribution of HMs in the sediments; 2) identify the composition of microbial communities in the sediments; 3) characterize differences in the distributions of MRGs; and 4) evaluate correlations between HMs, bacterial community compositions, and MRGs in the sediments of a river flowing through developing city. The results of this study provide a comprehensive understanding of the bacterial communities and MRGs in the sediments of an urban river and provide a theoretical basis for understanding the effects of HM pollution and environment risks in the presence of human development.

## 2. Materials and methods

### 2.1. Study area and sample collection

**Figure 1:**
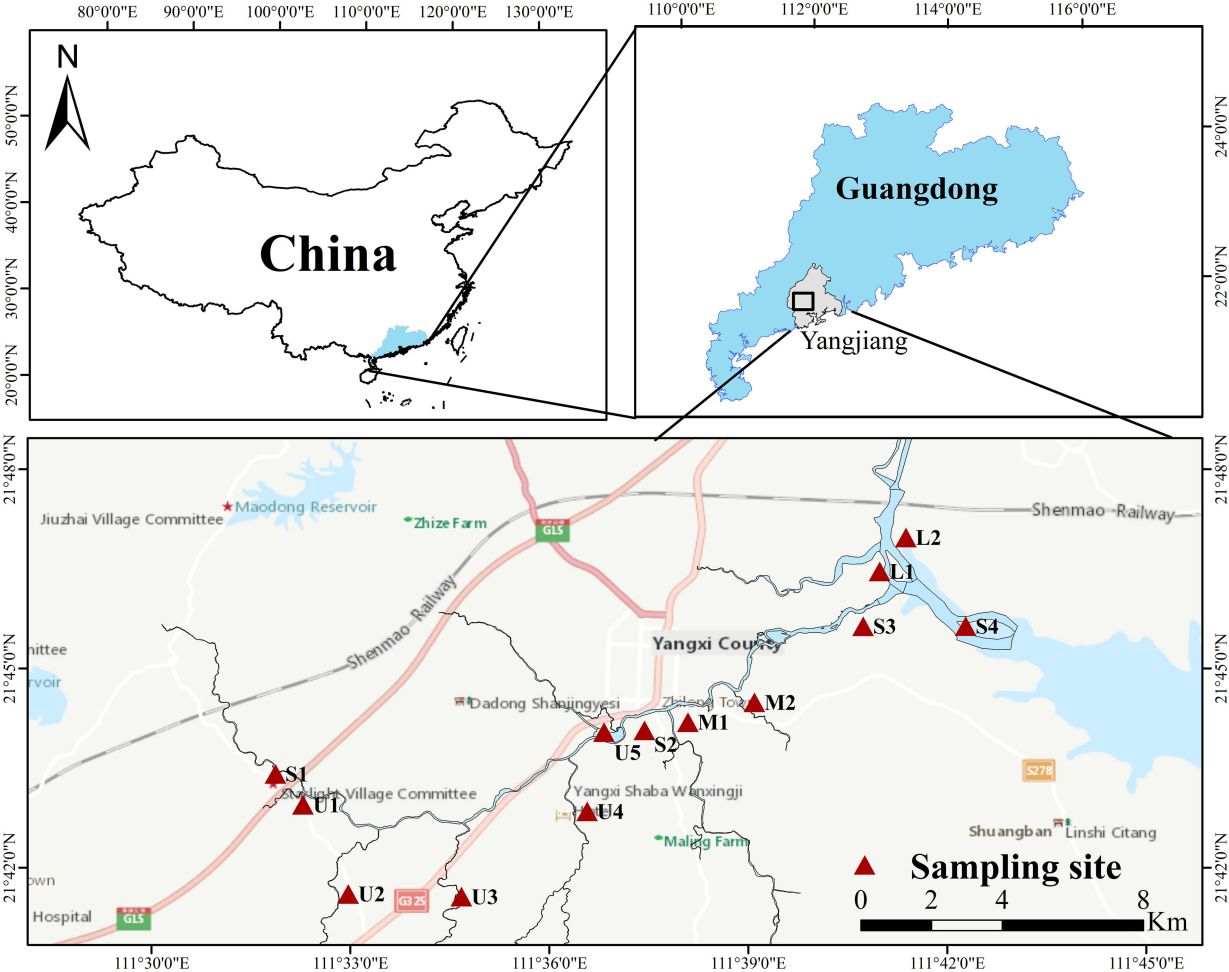
Location of water and sediment sampling section in the Zhilong River Basin in Yangjiang of Guangdong, China.

The Zhilong River basin was selected as the study area, near Yangjiang city in Guangdong Province, China. The Zhilong River is ~34.3 km long, with a drainage area of ~268 km^2^. The river flows through mainly farmland and countryside in the upstream areas, through the densely populated county center in the middle reach, and then flows downstream into the South China Sea along with the Fengtou River. Four mainstream and nine tributary samples were collected from the Zhilong River in September 2022 (Figure 1). Each sediment sample was composited from 3 to 4 replicates, which were collected using a Van Veen grab sampler from an approximately 2m^2^ area at each sampling station (Milakovic et al., 2020). Corresponding water samples were also collected at all sampling sites using a plexiglass 2-L water sample collector, and the total phosphorus (TP), ammonia nitrogen (NH_3_-N), and chemical oxygen demand (COD) were analyzed. The sediment samples were sealed in sterile bags and place in an incubator. After being transported to the laboratory, the samples of sediments were divided into two subsamples to analyze microbial indices and chemical concentrations. Subsamples used for determining HM concentrations and fractions, dissolved organic carbon (DOC), total nitrogen (TN), and total phosphorus (TP) were stored at 4°C. Subsamples used for metagenomic sequencing were stored at −80°C. Detailed information for the sampling sites is presented in Supplementary Table S1 and Figure 1, and the physicochemical characteristic of the water and sediments sampled from Zhilong River basin are shown in Supplementary Table S2.

### 2.2. Determination of heavy metals and their fractional distributions in sediments

Analysis of HM chemical fractions [cadmium (Cd), lead (Pb), nickel (Ni), Cu and chromium (Cr)] followed the Chinese Standards for Soil and Sediment—Sequential Extraction Procedure for Speciation of 13 Trace Elements (GB/T 25282-2010). Based on the method, the HMs were separated into four chemical fractions: the acid-soluble fraction (F1), reducible fraction (F2), oxidizable fraction (F3), and residual fraction (F4). Briefly, acetic acid and hydroxylamine hydrochloride solutions were used to extract the F1 and F2 fractions, respectively; hydrogen peroxide and ammonium acetate solutions were used to extract the F3 fraction; and a mixture of hydrochloric acid, nitric acid, hydrofluoric acid, and perchloric acid was used to extract the F4 fraction.

### 2.3. Metagenomic sequencing

DNA was extracted from the sediment samples using the cetyltrimethylammonium bromide (CTAB) method. The DNA concentration, degree of degradation, and potential contamination were measured using a Fragment Analyzer 5400 (Agilent, USA). Prior to DNA sequencing, an NEB Next^®^Ultra™ DNA Library Prep Kit from Illumina (NEB, USA) was used to construct a sequencing library following the manufacturer's recommendations. In brief, every DNA sample was sheared into DNA fragments of ~350 bp by sonication. The DNA fragments were then end-polished, A-tailed, ligated with a full-length adaptor for Illumina sequencing, purified, and amplified by polymerase chain reaction (PCR) to construct DNA libraries. After purification using an AMPure XP Kit, the quality of the constructed libraries was analyzed using an Agilent Bioanalyzer 2100 system and the amounts of DNA fragments were quantified using real-time PCR. Sequencing for library preparations was conducted using an Illumina NovaSeq 6000 platform and paired-end 150 sequencing. The generated sequencing datasets were deposited in the NCBI Sequence Read Archive database with a BioProject accession number of PRJNA947269.

### 2.4. Bioinformatics analysis

The raw data (paired-end reads) in the metagenomic datasets were filtered using Trimomatic v. 0.39 to remove sequencing adapters, reads with low-quality bases (default quality threshold value ≤ 20), and sequences with length <50 bp. The data were further filtered to eliminate host pollution using Bowtie2 v. 2.3.5.1 software (http://bowtie-bio.sourceforge.net/bowtie2/index.shtml) and then FastQC (http://www.bioinformatics.babraham.ac.uk/projects/fastqc/) was used to perform additional quality control. The species in the samples were identified using Kraken2, comparison software based on K-mer (Wood and Salzberg, 2014; Brum et al., 2015; Mandal et al., 2015; Lu et al., 2017) and a custom microbial database (including species sequences from the NT nucleic acid database and RefSeq whole genome database of NCBI). The relative abundances of the species in the samples were then predicted using Bracken. FMAP software was used to compare the clean reads with antibacterial biocide- and metal-resistance genes in BacMet (http://bacmet.biomedicine.gu.se/) (based on DIAMOND; Franzosa et al., 2018). Sequences were filtered out when not identified using default BacMet setting parameters (e-value <10^−3^ and percent identity > 80%). The relative abundances of potential resistance genes in each sample were represented as transcripts per million (TPM), calculated using the following equation (Zhao et al., 2020):


(1) $$\begin{array}{l} TPM\;=\;A\times \frac{1}{\Sigma \;A}\times 10^{6} \end{array}$$

(2) $$\begin{array}{l} \text{A =}\frac{total\;reads\;mapped\;to\;gene\;\times \;10^{3}}{gene\;length\;in\;bp} \end{array}$$

### 2.5. Heavy metal contamination and ecological risk assessment

The index of geoaccumulation (I_geo_), sediment quality guideline (SQG), and risk assessment coding (RAC) method were used to assess the levels of contamination and ecological risk of the HMs in sediments. I_geo_ and RAC are calculated as follows:


(3) $$\begin{array}{l} I_{\text{geo}}=log_{2}{\text{C}}_{\text{i}}/\left[k\times B_{\text{i}}\right] \end{array}$$

(4) $$\begin{array}{l} RAC=F1/HM\times 100\% \end{array}$$

where *C*_*i*_ is the concentration of HM *i* in the sediment sample, k is the variation due to diagenesis with a default value of 1.5 in this study, B_*i*_ is the background concentration of HM *i* in Guangdong province used as a default. RAC is the fraction of acid-soluble HMs, F1 is the concentration of acid-soluble HMs, and HM is the total concentration of all HMs.

HM concentrations were also compared to the Threshold Effect Levels (TELs) and the Probable Effect Levels (PELs) (Bai et al., 2014). The TELs for Cd, Pb, Ni, Cu, and Cr are 0.596, 35, 18, 35.7, and 37.3 mg·kg^−1^, respectively. The PELs for Cd, Pb, Ni, Cu, and Cr are 3.53, 90.3, 36, 197, and 90.0 mg·kg^−1^, respectively. Details of the grade standards and parameters for the above three assessment methods for HMs in sediments are provided in Supplementary Tables S3, S4.

## 3. Results

### 3.1. Concentrations, chemical fractions, and spatial distributions of heavy metals in sediments

**Figure 2:**
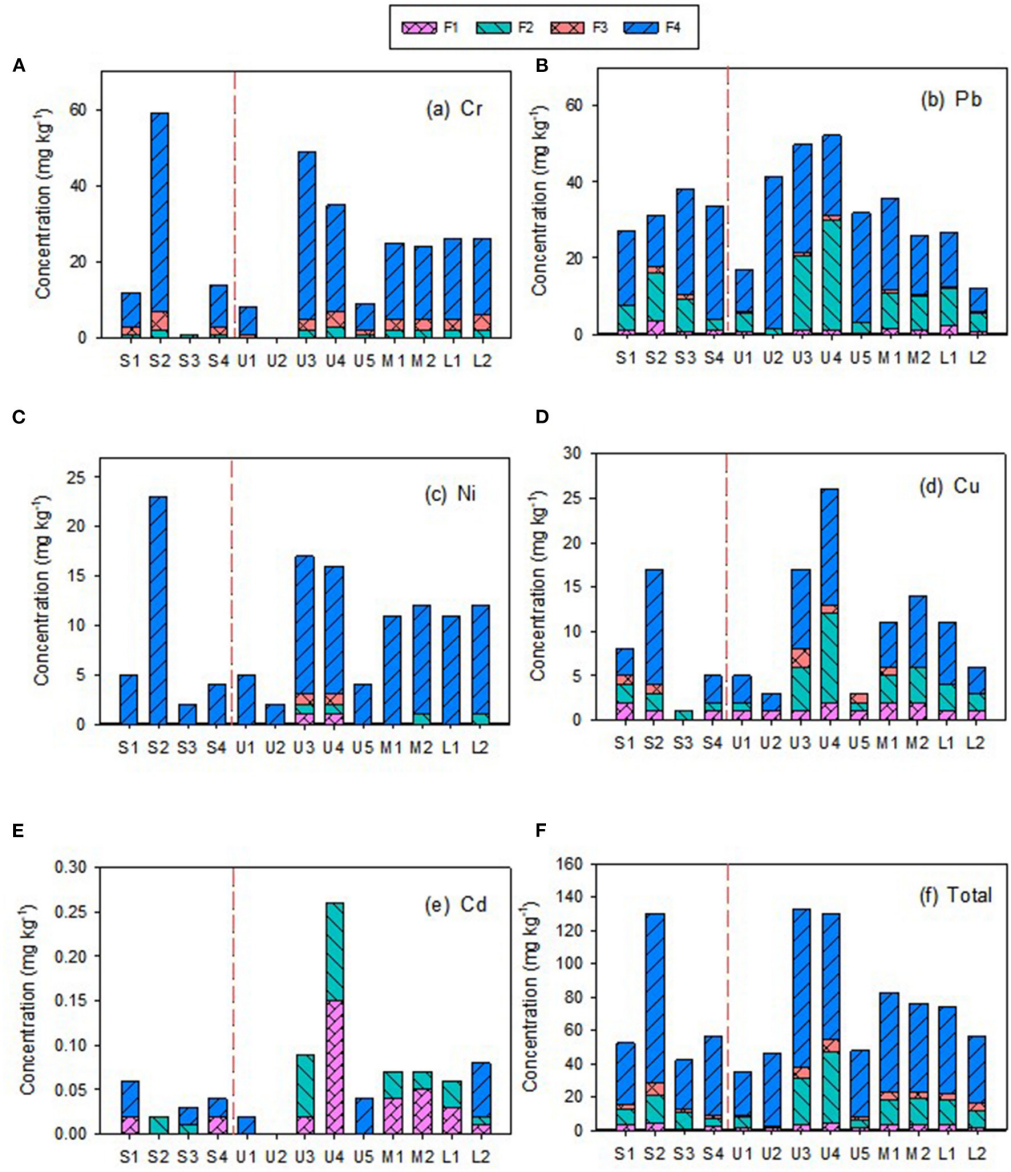
The chemical fraction concentrations of heavy metals in sediments sampled from the mainstream and tributary of Zhilong River Basin in Yangjiang of Guangdong, China.

The concentrations and distributions of HMs in the Zhilong River basin are shown in Table 1 and Figure 2. The concentrations of Cd, Pb, Ni, Cu, and Cr in sediment samples from the Zhilong River basin were similar to background values in Guangdong Province (Supplementary Table S4). The composition and concentrations of the four fractions (F1 to F4) of HMs in sediments that were extracted and analyzed in this study are shown in Figure 2. Cd and Cu were dominated by the mobile fractions (sum of F1 to F3) F1, with 57.0% and 52.3% in this fraction, respectively. These results indicate a high potential for ecological risk caused by deposition and dispersion of the mobile fraction. Pb and Cr also had relatively high proportions of this fraction, 34.3% and 23.6%, respectively. Only Ni had a low proportion in the mobile fraction (4%), which indicated low potential ecological risk.

**Table 1:** Heavy metal concentrations in sediments sampled from Zhilong River Basin in Yangjiang of Guangdong, China.

**Index**	**Heavy metal (mg kg** ^**−1**^ **)**					
	**Cd**	**Pb**	**Cu**	**Ni**	**Cr**	**Total**
**Mainstream**						
Min	0.010	28.6	0.500	1.50	2.00	46.6
Max	0.050	42.6	20.0	26.0	59.0	136
Average	0.040	34.1	8.38	9.63	21.2	73.4
Standard deviation	0.020	6.04	8.34	11.1	25.6	42.3
CVs (%)	49.5	17.7	99.6	115	121	57.7
**Tributary**						
Min	0.010	13.2	3.00	3.00	2.00	35.7
Max	0.25	51.4	24.0	21.0	50.0	140
Average	0.080	33.2	10.6	11.1	22.7	77.6
Standard deviation	0.07	13.5	7.07	6.03	14.5	35.5
CVs (%)	93.7	40.6	67.0	54.3	64.0	45.8

Results for HM concentrations and ecological risk in sediments as represented by I_geo_, RAC, and SQGs are summarized in Supplementary Table S5. For I_geo_, most HMs were <0, indicating that there was little HM contamination in the studied basin. However, the I_geo_ for Cd at station U4 was in the range of uncontaminated to moderately contaminated (0 <*I*_*geo*_ ≤ 1), The results of the RAC method showed moderate risk to very high risk due to Cd and Cu in about 61.5% of the samples, and no risk to low risk due to Ni, Cr, and Pb in most of the studied samples (84.6–100%). For the SQG analysis, only 15.4, 23.1, and 38.5% of Cr, Ni, and Pb concentrations in sediments, respectively, were between TELs and PELs, representing moderate risk. Concentrations of these HMs in other samples and Cd and Cu in all samples were lower than the TELs, indicating no or low risk. These results suggest that although the degree of pollution of these HMs in the study area was relatively low, they nevertheless presented moderate ecological risks.

To analyze the spatial distributions of the HMs, the mainstream samples (S1–S4) and tributary samples (upstream U1–U5, midstream M1–M2, lower L1–L2) were evaluated separately; the corresponding concentrations of HMs are presented in Figure 2. The concentrations of individual and total HMs in mainstream sediments were generally lower than those in tributary sediments, implying source influences from tributary inflows. The concentrations of TOC, TN, and TP in tributary sediment samples were also clearly higher than those in the mainstream sediment samples (Supplementary Table S2). However, the coefficients of variation (CVs) for TOC, TN, and TP in the mainstream samples (37.4–61.5%) were similar to those for tributary samples (39.6–73.5%) and the CVs for the five HMs in mainstream samples (17.7–121%) were similar to those in the tributary samples (40.6–93.7%), indicating similar variations in these parameters between mainstream and tributary samples. Overall, these results suggest that the tributaries are important sources of HMs and nutrients to the mainstream sediments.

Higher concentrations of Cu, Ni, and Cr were found in the S2 sample from mainstream sediments (Figure 2). Cr, Cu, and Ni concentrations decreased from S2 to downstream (S3 and S4), particularly S3. However, the concentrations in the tributaries were not similarly distributed in the corresponding M1, M2, L1, and L2 samples, suggesting that these tributaries were not the main source of these HMs in the downstream river sediments. Variations in Pb concentrations were relatively small in both mainstream and tributary samples. Cd had the lowest concentrations of the five investigated HMs and also had low variability, except for the U4 sediment sample, which had a relatively high concentration (Table 1 and Figure 2). Moreover, except for Cd in the U4 sample, Cd and Pb exhibited relatively homogeneous distributions within the watershed (Table 1 and Figure 2).

### 3.2. Profiles and spatial distributions of the microbial community in sediments

**Figure 3:**
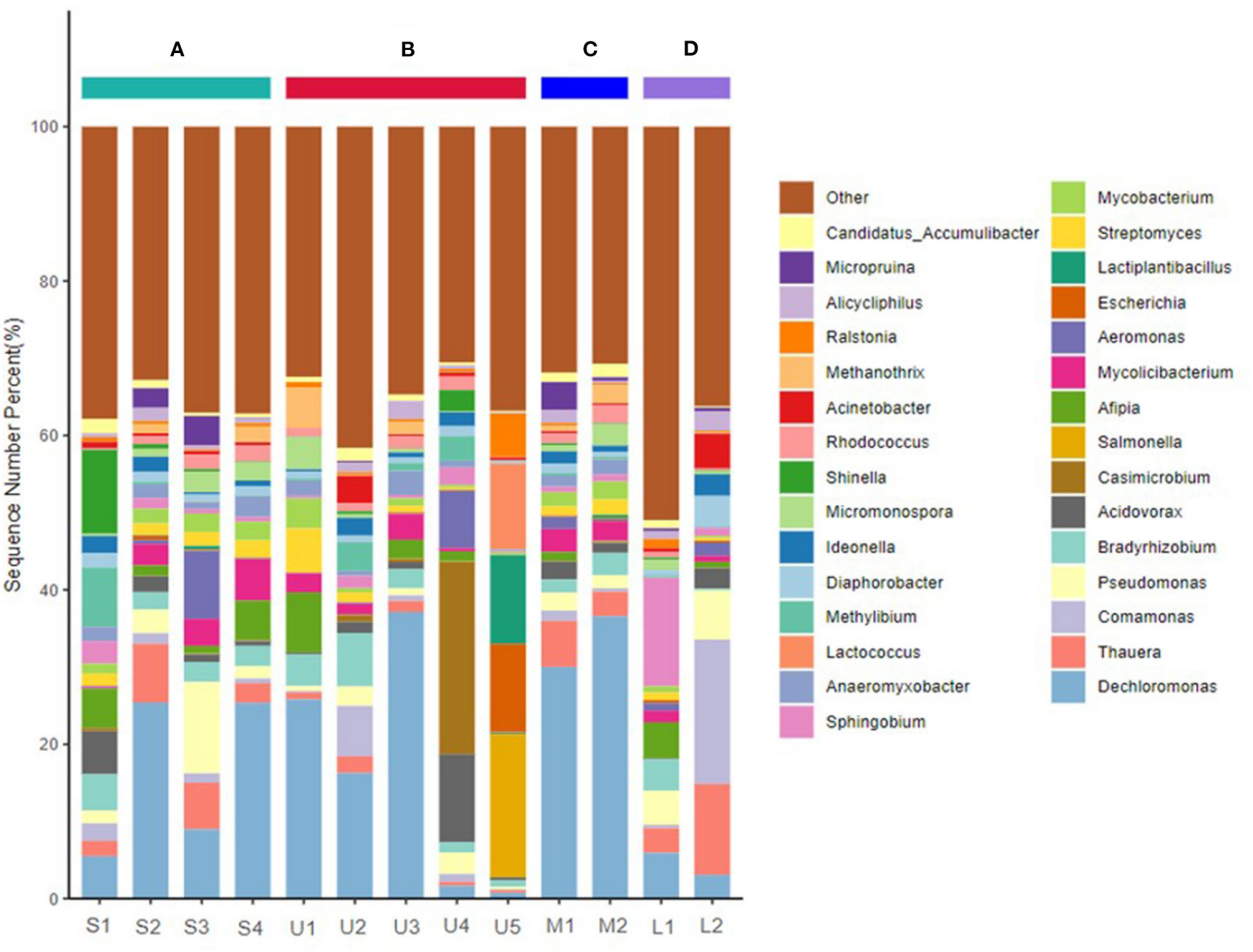
Taxonomic composition of microbial communities of sediments sampled from main stream and tributary of Zhilong River basin at the genus level. **(A–D)** Represent the mainstream and tributaries in the upper, middle, and lower streams, respectively.

After filtering, 20,212,665–22,966,333 sequences from raw reads were obtained from the 13 samples. The most frequently identified species were Bacteria, Archaea, Fungi, Heunggongvirae, and Viruses, comprising 97.80, 1.42, 0.45, 0.26, and 0.07%, respectively. Proteobacteria (51.60–87.22%), Actinobacteria (6.40–22.94%), Firmicutes (1.03–37.29%), Acidobacteria (0.09–5.88%), Euryarchaeota (0.08–7.55%), Cyanobacteria (0.10–5.06%), and Nitrospirae (0.09–3.25%) were the most abundant phyla in the Zhilong River sediments (Supplementary Figure S5). The 30 most dominant bacteria accounted for 49.01–69.47%, among which the most abundant genus found in all the samples were *Dechloromonas* (0.76–37.14%), *Thauera* (0.36–11.79%), *Comamonas* (0.12–18.70%), *Pseudomonas* (0.31–11.83%), *Bradyrhizobium* (0.25–6.92%), and *Acidovorax* (0.13–11.41%) (Figure 3), mainly affiliated with Proteobacteria.

The bacterial composition varied spatially along the mainstream, with stronger variations observed in river sediments in the tributaries, consistent with the results of the HM analysis. Proteobacteria abundance decreased downstream in the Zhilong River, while Actinobacteria and Fimicutes increased downstream. In addition, the coastal tributaries showed obvious differences in microbial communities. For examples, compared with the mainstream sample S2, more Proteobacteria and less Actinobacteria were detected in upstream tributaries at samples U2 and U4, respectively. U5 had a particularly high content of Fimicutes. Compared with the mainstream sample S3, clearly higher contents of Acidobacteria were present in the downstream tributary samples M1 and M2. Thus, areas near samples U2, U4, U5, M1, and M2 may be the main sources to the mainstream of these microbial communities.

Spatial variations in the bacterial compositions at the genus level were greater than those at the phylum level (Figure 3 and Supplementary Figure S5). The dominant genus at Station S1 were *Dechloromonas, Acidovorax, Afipia, Methylibium*, and *Shinella*, all with abundance >5%. However, their dominance diminished in sediments from the midstream and downstream areas. *Dechloromonas* was a major contributor among all genus found at Stations S2–S4, with a nearly 5-fold increase at Stations S2 and S4. *Pseudomonas* (11.83%) was found as the dominant genus only at Station S3. The microbial community structure varied greatly along the river and obvious differences in genus composition were also found in the tributaries. For example, *Dechloromonas* was the dominant genus at Stations U1, U2, U3, M1, M2, and L1. Station U4 was dominated by *Acidovorax* and *Casimicrobium*; relatively high abundances of *Salmonella, Escherichia, Lactiplantibacillus*, and *Lactococcus* (18.45%, 11.35%, 11.51%, and 11.11%, respectively) were present at Station U5. *Sphingobium* was the dominant genus at Station L1, but *Thauera, Comamonas*, and *Pseudomonas* had higher abundances at Station L2.

Correlation analysis was used to evaluate the influence of microbial composition and structure in the tributaries on the mainstream. Significant correlations in genera abundance (*R* = 0.837–0.958, *p* < 0.01) were found among the middle and lower mainstream stations S2–S4 (Supplementary Table S6), suggesting similar microbial community structure compositions in these areas. Strong correlations (*R* = 0.470–0.946, *p* < 0.01) among microbial community structure compositions were also observed between these mainstream sediments and some upstream and midstream tributary stations (U1–U3, M1, and M2) (Supplementary Table S6). However, differences in the compositions of microbial communities between Station S4 and downstream tributary Stations L1 and L2 were also observed (Figure 3), implying little influence of the downstream tributary on sediments at Station S4.

### 3.3. MRG distributions in sediment from the Zhilong River basin

In sediment samples from this study, 294 MRGs (belonging to 16 single-MRG types and 46 multi-MRG types) were annotated. MRGs for As, Zn, and Cu were the dominant single-MRG types with high abundance (> 20%), followed by Ni, Fe, Cr, W, Hg, and Ag with moderate abundance (1.00–11.0%). Other single-element MRGs (e.g., Cd and Pb) had low abundance (<0.5%) (Supplementary Figure S6). Although Cu, Ni, Cr, Cd, and Pb all had low concentrations in sediments in this area, the abundances of these single-MRGs varied greatly within the sediment samples. The 20 MRGs with the highest abundance, including multimetal, As, Cu, and Zn MRGs, together accounted for 58.0–63.7% of all MRGs in the sediment samples, most of which were significantly correlated (*p* < 0.01) (Supplementary Figure S7), suggesting that the dominant MRGs in the collected sediment samples were similar.

**Figure 4:**
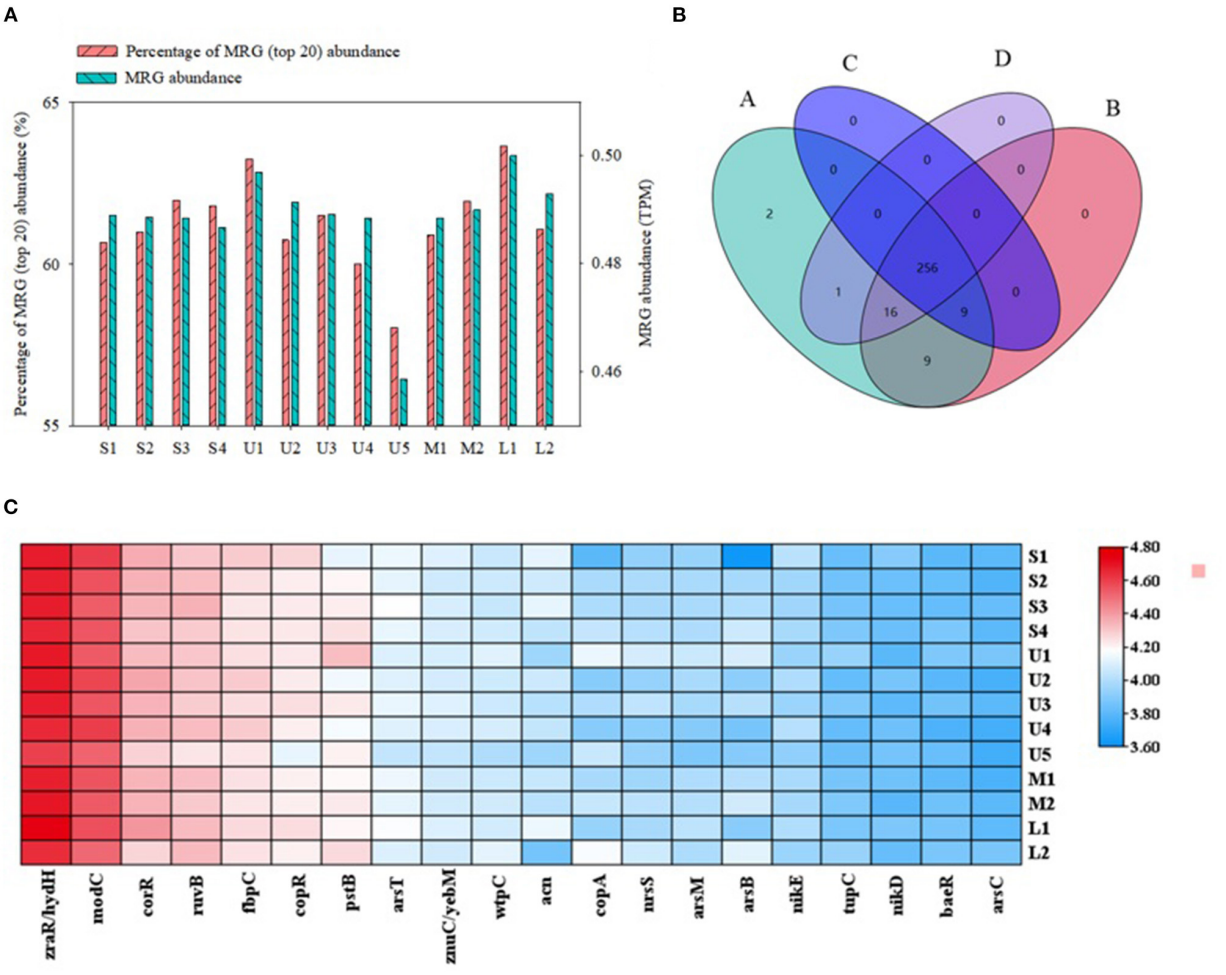
The characteristics of metal resistance genes (MRGs) subtypes in sediment samples: **(A)** The abundance distributions of MRGs subtypes and percentage of MRG (top 20) abundance (based on method of transcripts per million, TPM), **(B)** Four-set Venn diagram of MRG subtypes [**(A)** represent the mainstream S1-S4, and **(B–D)** represent tributary of upstream U1-U5, midstream M1-M2, lower L1-L2, respectively], and **(C)** heatmap of metal resistance genes (MRG) subtypes about five metals.

Except for U5, the 20 MRGs with the highest abundance generally presented similar spatial distributions in samples collected from the mainstream and tributary sediments (Figure 4A); no significant differences were found among sediments from upstream, midstream, and downstream tributaries (*p* > 0.05) (Figure 4C). A Venn diagram for these samples shows that 87.4% of the MRGs co-occurred in the mainstream and three groups of tributaries; only two MRGs were unique to the mainstream (Figure 4B). The 20 most abundant MRGs in the sediment samples were also significantly correlated among the stations (Supplementary Table S7). Thus, the distributions of the dominant MRGs in this region had only small spatial differences. This could be attributed to relatively stable abundances of metal resistance bacteria, which act as MRG carriers.

For certain low-abundance MRGs, there were large differences in abundance between mainstream and tributary samples, and significant differences were also found among tributaries. For example, the *Silc, TerD*, cop-unnamed, and *CopF* subtypes in mainstream samples were significantly more abundant than in tributary samples. The merF and merP subtypes were more abundant in upstream than in downstream tributary sediments, whereas the *silA, terD*, and *zntR*/*yhdM* resistance genes were more abundant in downstream sediments (Supplementary Figure S7). These results may be due to the low abundances of metals in sediments of the Zhilong River in this study (Yin et al., 2017).

### 3.4. Correlations between MRGs, the microbial community, and environmental factors

**Figure 5:**
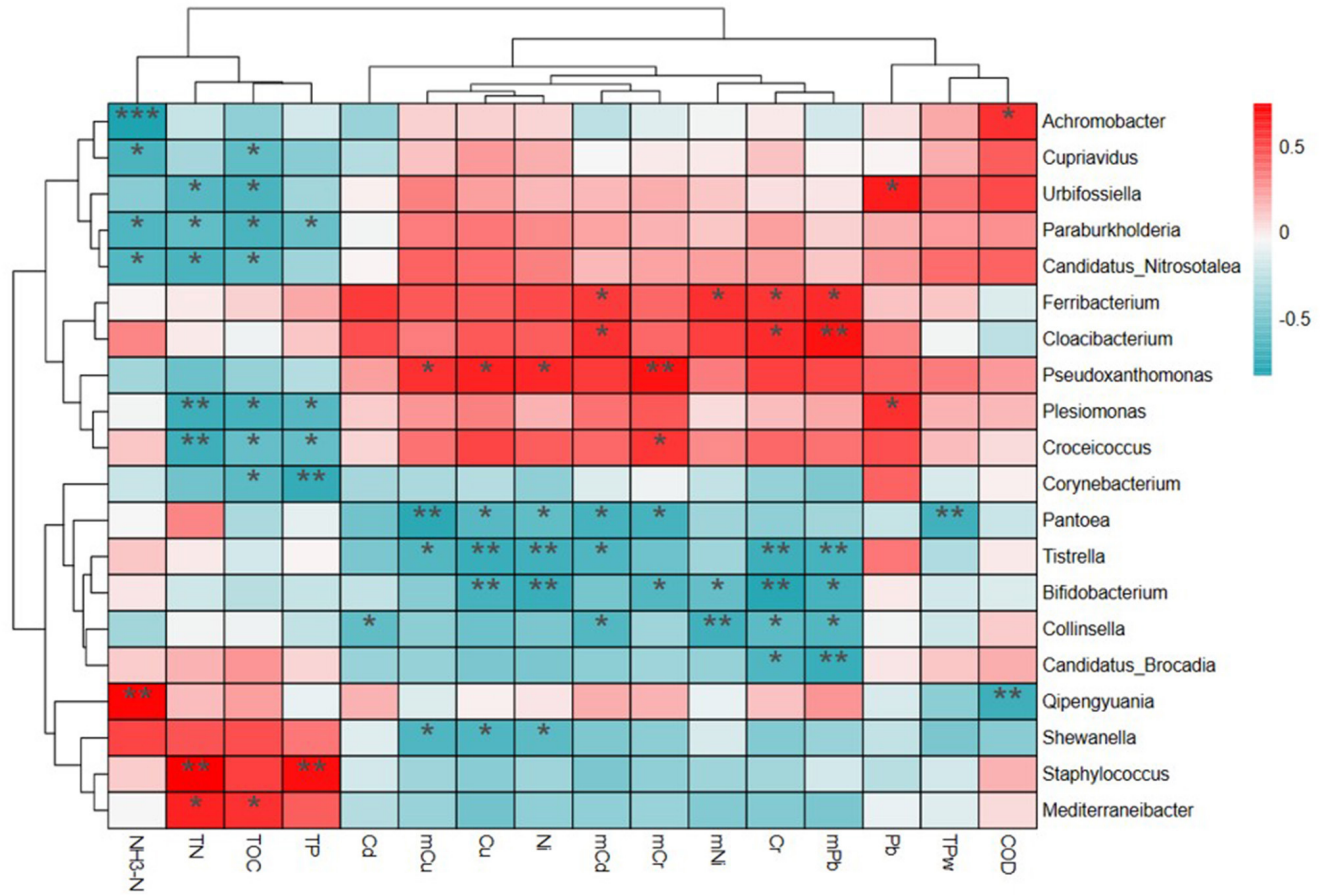
The relationship of bacterial communities at the genus level and environmental pollutants based on the heatmap and cluster analysis.

Figure 5 shows correlations between environmental factors and microbial communities at the genus level, based on the 20 most relevant features. Four clusters of genus and three clusters of environmental factors were identified using phylogenetic trees. The genus *Achromobacter, Cupriavidus, Urbifossiella, Paraburkholderia*, and *Candidatus*_*Nitrosotalea* in Cluster 1 and *Plesiomonas, Croceicoccus*, and *Corynebacterium* in Clusters 2 and 3 had significant negative correlations (*p* < 0.01) with NH_3_-N, TN, TOC, and TP. However, significant positive correlations were found between these indexes and *Qipengyuania, Shewanella, Staphylococcus*, and *Mediterraneibacter* in Cluster 4. There were significant negative correlations between the genus *Pantoea, Tistrella, Bifidobacterium, Collinsella*, and *Candidatus_Brocadia* in Cluster 3 with five of the HMs. However, positive correlations were observed between the microbial communities (*Ferribacterium, Cloacibacterium, Pseudoxanthomonas, Plesiomonas*, and *Croceicoccus*) and HMs in Cluster 2.

**Figure 6:**
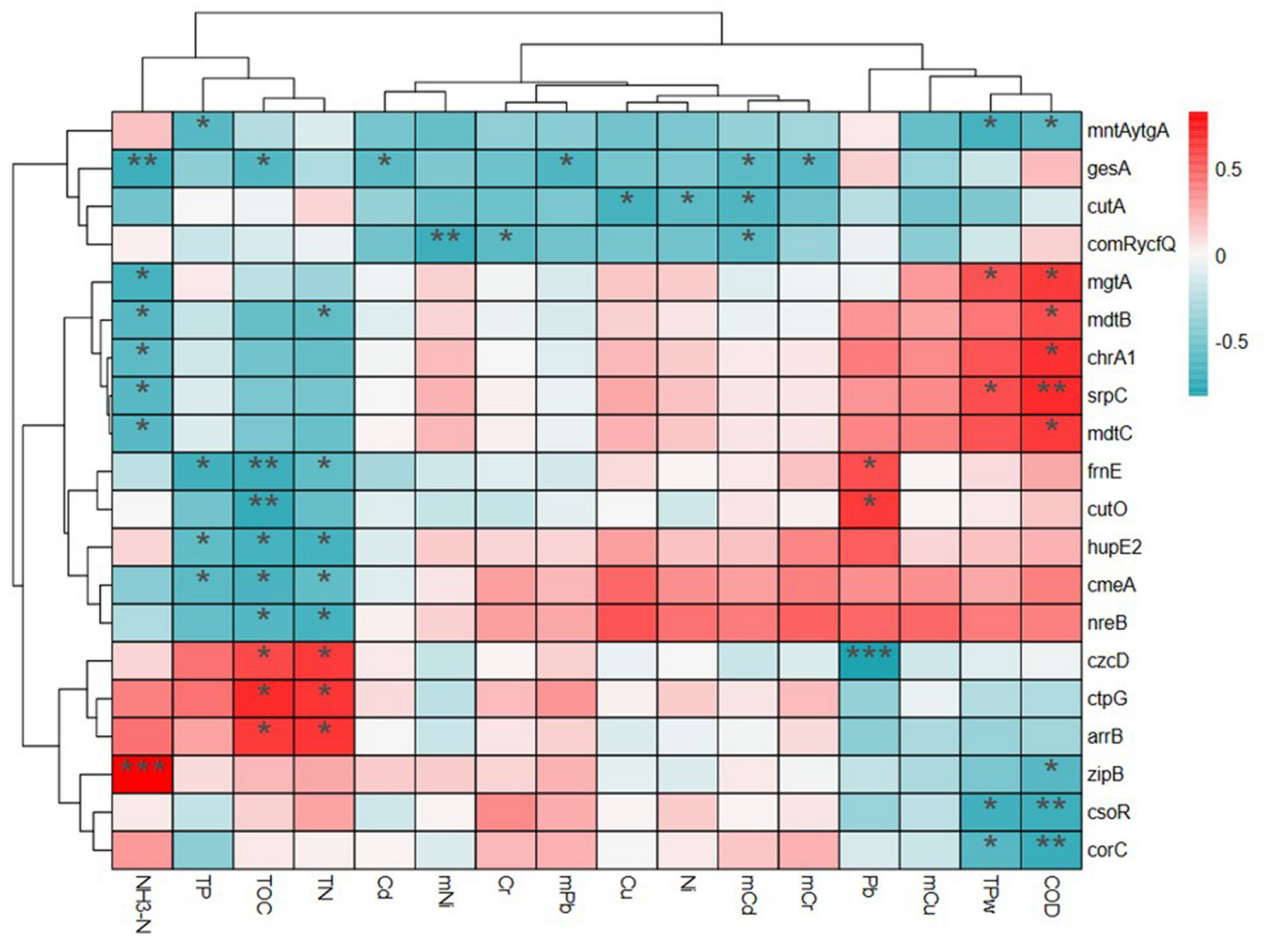
The relationship of metal resistance genes (MRGs) and environmental pollutants based on the heatmap and cluster analysis.

Correlations among MRGs and environmental factors were also analyzed using heatmap and cluster analysis and four clusters were presented in the results (Figure 6). In Group 1, the relative abundances of MRG subtypes *mntAytgA, gesA, cutA, comRycfQ* were negatively correlated with environmental factors. For Group 2, mgtA, mdtB, chrA1, *srpC*, and *mdtC* were negatively correlated with NH_3_-N in water, but positively correlated with TP and COD in water. For Group 3, *frnE, cutO, hupE2, cmeA*, and *nreB* were significantly positively correlated with TP, TOC, and TN in sediments and frnE and *cutO* were significantly correlated with Pb. In all four groups, *czcD, ctpG*, and *arrB* were positively correlated with TOC and TN in sediments and *zipB, csoR*, and *corC* were positively correlated with TP and COD in water. The heatmap also showed that *czcD* was correlated with Pb and *ZipB* was correlated with NH_3_-N.

**Figure 7:**
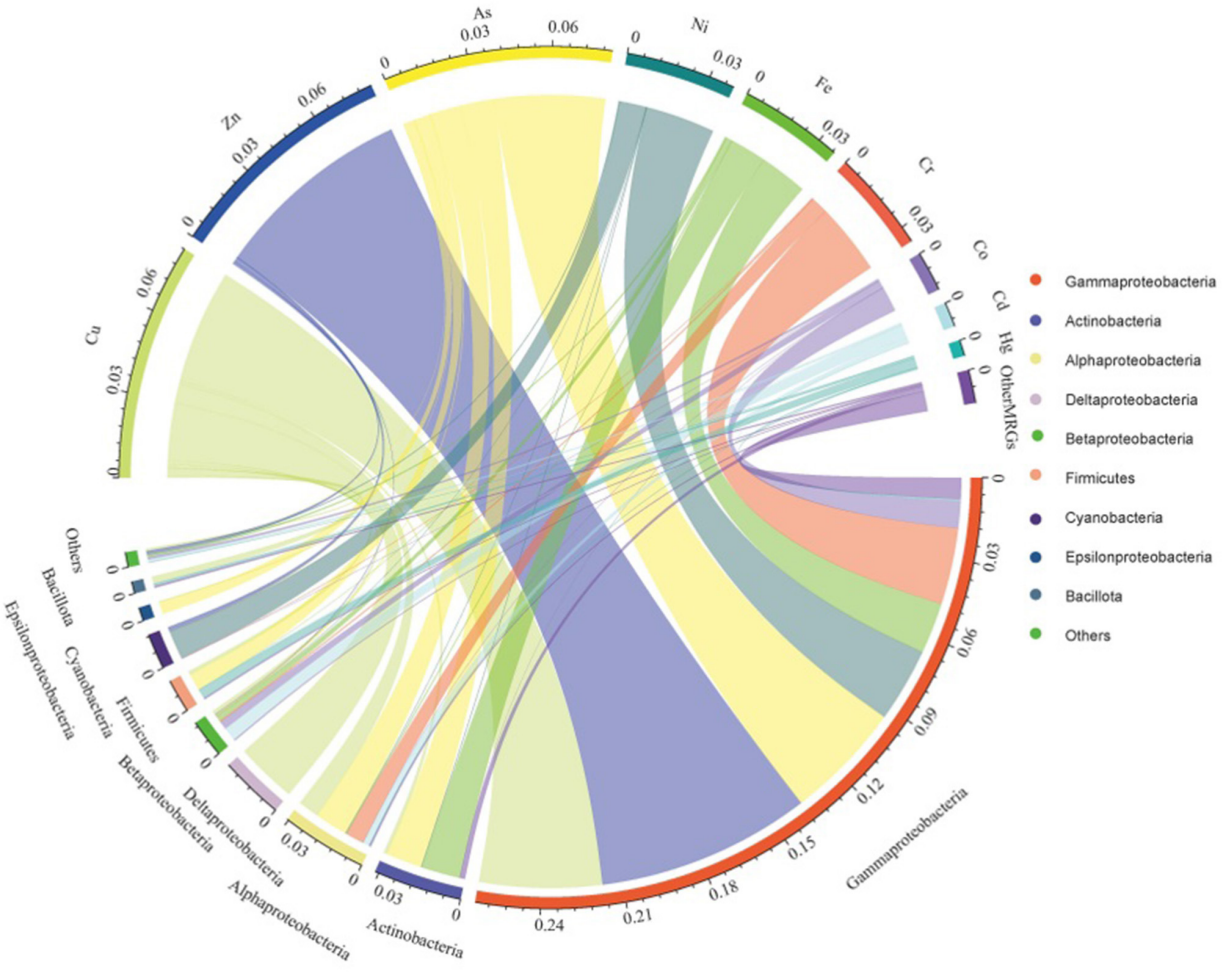
Categorization of MRGs and potential bacterial hosts according to metals and bacterial taxonomy at the phylum or subphylum level in the sediments.

Relationships between potential bacterial hosts and corresponding MRGs in the sediments are shown in Figure 7. Cu, Zn, As, Ni, Fe, and Cr MRG abundances were mainly correlated with the expected hosts such as Gammaproteobacteria, Actinobacteria, Alphaproteobacteria, and Deltaproteobacteria, based on metals concentrations and bacterial taxonomy at the phylum or subphylum level. The dominant predicted hosts of different single-MRG types differed in the sediment samples. For example, the hosts of Cu MRGs were mainly Gammaproteobacteria (54.2%), Deltaproteobacteria (27.0%), and Alphaproteobacteria (9.24%). Gammaproteobacteria (70.6%), Firmicutes (64.6%), Betaproteobacteria (59.9%), and Cyanobacteria (27.1%) were the dominant hosts of Hg, Cd, and Ni MRGs.

## 4. Discussion

### 4.1. Concentrations and spatial distributions of heavy metals in sediments

Compared to HM concentrations in sediments from other rivers of China (Supplementary Table S8), average Pb concentrations in this study were higher than in the Ganjiang River (Shi and Zhang, 2019), Daqing River (Tang et al., 2015), and middle reach of the Yarlung Zangbo River, but lower than those of Lower Lijiang River (Xiao et al., 2021), Maozhou River (Gong et al., 2016), Lower Yangtze River (Wang et al., 2018), Xiaoqing River (Jiao et al., 2017), and Huangshui River (Bai et al., 2014). The Cr concentrations in this study were lower than in the above rivers, except for the Ganjiang River. The Ni concentrations were higher than in the Ganjiang River and Lower Yangtze River, but were lower than in the other rivers. The Cu and Cd levels were relatively low compared to those in other rivers in China. Overall, HM concentrations in the Zhilong River were low compared to other rivers in China.

The moderate risk associated with HMs in this study can be attributed to their chemical fractionation. Generally, F1 is considered the bioavailable fraction and the sum of F1, F2, and F3 represents the mobile fraction (Rauret et al., 1999); thus, a high percentage of F4 indicates minor ecological risk to biota. The higher percentages of F1–F3 in this study indicate potential ecological risks associate with these HMs, despite their relatively low concentrations.

The influence of tributaries on HM distribution in the mainstream and spatial differences in HM concentrations among the tributaries may be due to differences in sources and hydrology. HMs were found to be unevenly distributed upstream, likely related to tributary Stations U3 and U4 with higher concentrations and flows than the other tributaries (Supplementary Figure S1). This could be one of main contributors of HMs at Station S2, located in middle reach, which also receives discharged wastewater from the county. The F1 fraction of HMs in sediment determined by the positive matrix factorization (PMF) model (Supplementary Figure S2) can be interpreted as anthropogenic sources. The F1 fraction mainly comprised Cd, Cu, and Ni, which could be related to discharge of contaminants in domestic wastewater. The CVs for Cu, Ni, and Cr were similar, which may indicate a similar source to the sediments (Table 1). The Pearson correlation analysis identified significant linear correlations among these HMs (*p* < 0.01, 0.70 <*R* < 0.79) (Supplementary Figure S3). In the downstream area, the tributary (Station L2) discharges cleaner sediments (Supplementary Figure S1), which may account for lower levels of these contaminants at S4. TP and COD were decreased downstream along the river, also suggesting that dilution plays an important role in reducing HM concentrations (Supplementary Figure S4). Pb may originate from natural sources, consisted with the source analysis indicating that F1 was strongly correlated with Pb (Supplementary Figure S2). The lowest concentration of Pb was found at Station L2, potentially related to high water flow and velocity (Supplementary Figure S1). A significant correlation was found between Cd and TP (*p* < 0.01, *R* = 0.693), a primary contaminant from phosphate fertilizer, consistent with the source analysis for F2 (Supplementary Figure S2). Moreover, regional differences in lithologic inputs, geologic features, hydrologic characteristics, and vegetation cover may also have influenced spatial variations in HM concentrations in this study (Omwene et al., 2018).

### 4.2. Occurrence and spatial distributions of microbial communities and MRGs

Although HM concentrations were low in this study, the dominant microorganisms were consistent with the results of previous studies in areas with high concentrations of HMs. Proteobacteria, Acidobacteria, and Firmicutes were found to be the dominant phyla in sediment assemblages from e-waste affected rivers (Liu et al., 2018). Proteobacteria, Bacteroidetes, and Firmicutes were the core functional phyla found in river sediments with long-term high concentrations of HMs (Chen et al., 2018). Firmicutes are particularly active in HM-contaminated sediments (Jacquiod et al., 2018). The similarity of these communities of dominant microorganisms could be related to their varying sensitivity to HM concentrations (Chen et al., 2019). For example, in a previous study, Acidobacteriota were found to have lower abundance at sites with higher concentrations of HMs; however, the opposite trend was found for Cyanobacteria (Gupta et al., 2022).

Aside from HMs, other anthropogenic contaminants could account for the microbial diversity and biomass observed in sediments. *Dechloromonas* demonstrated a clear positive correlation with Cu, Cr, Cd, and Pb in aquaculture sediments (Xu et al., 2022b), but was dominated by denitrifiers and their abundance was significantly affected by anthropogenic pollution (Guan et al., 2022). Among these bacteria, *Thauera* is an important genus with metabolic versatility used to remediate environmental pollutants in wastewater treatment (Zhang et al., 2020). *Comamonas sp*. was isolated from a Milma dairy effluent sample (Prabisha et al., 2015). Previous studies have shown that many validated species of genus *Pseudomonas* isolated from metals-contaminated area have the ability to counteract trace metal pressure via various resistance mechanisms (Cánovas et al., 2003; Huang et al., 2016). *Bradyrhizobium* has been extensively identified as biologically important in soils, performing metabolic functions of nitrogen fixation in symbiosis with plants, denitrification, aromatic compound degradation, and photosynthesis (Jin et al., 2022). The genus *Acidovorax* of *Betaproteobacteria* and the genus *Pseudomonas* of Gammaproteobacteria have important denitrifying functional genes (*nirS, nirK*, and *nosZ*) and have frequently been detected in areas with high groundwater denitrification (Xu et al., 2022a).

The above-mentioned dominant microorganisms have frequently been found as the main hosts of HMs in previous studies. Many bacterial species carrying MRGs are also the dominant bacterial species in sediments, which may result in the ability to adapt to metal stresses. *Proteobacteria* are ubiquitous microorganisms in the environment and are widely distributed in sediment (Pang et al., 2016; Yang et al., 2016; Kumar et al., 2019; Zhang et al., 2019). *Proteobacteria* are carriers of various MRGs and play a pivotal role in their transmission (Guo et al., 2019). Firmicutes members have also been found to possess various MRGs (Chen et al., 2018). Cyanobacteria and Actinobacteriota are known to be major antibiotic-producing bacteria and are often associated with multiple antibiotic resistance (Hu et al., 2017).

In addition, hydraulic conditions can indirectly affect the distribution of microbial communities through modifying HM concentrations and microbial community structure (Chen et al., 2020). The relatively high flow velocity and volume at Station S4 (Supplementary Figure S1) may explain differences in the microbial communities between downstream Station S4 and nearby tributaries (Supplementary Table S6).

Tributaries as the primary external sources of immigration strongly affect the genus structure in the mainstream, consistent with a previous study (Wu et al., 2022). In addition, differences in the Alpha, Shannon, and Simpson diversity indexes for the microbial communities between mainstream and tributary (Supplementary Table S9) were insignificant (*p* > 0.05). Thus, although the influence of the tributaries may partially explain variations in the profiles and distribution of HMs, microbial communities, and MRGs in the mainline sediments to some extent, effects of the dominant tributaries on the mainstream vary. For example, HMs at Station S2 were mainly affected by inputs from Stations U3 and U4; microorganisms were closely related to those at Stations U1, U2, and U3; while MRGs had similar compositions in all areas.

### 4.3. Correlations among environmental factors, microbial communities, and MRGs

MRGs were widely distributed among microbial genomes across the different sample types (Pal et al., 2015; Li et al., 2017). The succession of microbial communities was the main factor driving the overall profile of the MRGs (Song et al., 2019; Xie et al., 2019). Thus, the presence of MRGs in sediments was affected by changes in microbial communities to some extent. In addition, the degree of HM contamination affects MRG abundance (Wang et al., 2023), and different microbes may up- or down-regulate MRGs at similar levels of contamination (Song et al., 2019; Gupta et al., 2022). Therefore, as the common hosts of MRGs, bacteria can directly affect MRG abundance, potentially counteracting up-regulation or down-regulation due to environmental factors. The combination of these effects may explain the similar profiles and distribution of MRGs throughout the Zhilong River.

While the bacterial community and environmental contamination are important factors shaping MRG profiles in the environment (Di Cesare et al., 2016; Yang et al., 2018), mobile genetic elements are also important in propagating MRGs (Wright et al., 2008). For example, the recombinase *intI1*, a mobile genetic element, has been used as a proxy indicator for anthropogenic pollution (Gillings et al., 2015). It has been found to be significantly correlated with MRGs in municipal wastewater treatment plants in areas of intense human activity (Di Cesare et al., 2016). In this study, correlations were only found between the Ag and V MRGs and *intI1* (Supplementary Figure S8), indicating that *intI1* had the primary influence on MRG propagation for these metals rather than the individual MRGs for the five HMs investigated in this study. Thus, anthropogenic pollution likely had some contribution to the distribution of MRGs in the river, but acted mainly through multi-MRGs. These analyses also suggest that bacterial composition, environmental factors, and mobile genetic elements jointly affected the MRG profile in the Zhilong River.

## 5. Conclusion

The tributaries were the main contributors of conventional pollutants to the mainstream and the profiles of the pollutants were related to their sources. The profiles of HMs and bacterial communities in this study had significant differences in spatial distribution, with widely varying influences from the tributaries on the mainstream. There were large spatial variations in HMs among mainstream and tributary sediments. HMs in the tributaries had a strong effect on upstream areas, while the bacterial communities in the tributaries strongly affected both upstream and midstream areas. Compared to HMs and bacterial communities, the MRGs in sediments showed greater consistency in spatial distribution, potentially attributable to the combined effects of multiple contaminants, contaminant sources, differing chemical fractions, and hydrological effects rather than direct reflecting HM contamination and bacterial communities. The profiles of the HMs, bacterial communities, and MRGs in these low-contamination river sediments were significantly different in spatial distribution from one another.

## Data availability statement

The datasets presented in this study can be found in online repositories. The names of the repository/repositories and accession number(s) can be found below: NCBI - PRJNA947269.

## Author contributions

LF: methodology, data analysis, investigation, and writing—original draft. YY: project administration, formal analysis, and resources. FY and JX: sampling and water quality parameters analysis. HF and WL: project administration. ZX: modification. FZ: resources. SL: supervision, writing—review and editing, funding acquisition, and validation. All authors contributed to the article and approved the submitted version.
