# Supplementary material for: Profiles and spatial distributions of heavy metals, microbial communities, and metal resistance genes in sediments from an urban river
Source: Front Microbiol. 2023 Jun 29;14:1188681. doi: 10.3389/fmicb.2023.1188681 (PMC10340544; doi:10.3389/fmicb.2023.1188681)
Supplement: Supplementary file 1 [file Data_Sheet_1.docx]

Supplementary Information

**Profiles and spatial distributions of** **heavy metals, microbial communities, and metal resistance genes in sediments** **from an urban river**

**Lingfang Fu, Yang Yu, Fei Yu,** **Jieer Xiao, Huaiyang Fang, Weijie Li, Zhijie Xie, Feng zhang, Shu Lin***

*** Correspondence:** Shu Lin: linshu@scies.org

**Table S1.** Detailed information and physical characteristic of monitoring sites in the Zhilong River basin in the Yangjiang city in south China.

| Monitoring station | Longitude  (°E) | Latitude  (°N) |  | | Water (mg L^-1^) | | | | | | |  | Sediment (mg kg^-1^) | | | | |
| --- | --- | --- | --- | --- | --- | --- | --- | --- | --- | --- | --- | --- | --- | --- | --- | --- | --- |
|  |  |  |  | | TP | | COD | | | NH_3_-N | |  | TOC | TN | TP | | |
| *Mainstream* | | | | | | | | | | | | | | | |  |  |
| S1 | 111.5226 | 21.72594 | |  | | 0.20 | | 25 | 0.14 | |  | | 3.78 | 0.096 | 0.23 | |  |
| S2 | 111.6165 | 21.73702 | |  | | 0.090 | | 21 | 0.30 | |  | | 6.96 | 0.27 | 0.40 | |  |
| S3 | 111.6723 | 21.76350 | |  | | 0.050 | | 16 | 0.58 | |  | | 11.5 | 0.22 | 0.50 | |  |
| S4 | 111.6984 | 21.76353 | |  | | 0.00 | | 14 | 0.42 | |  | | 2.96 | 0.26 | 0.17 | |  |
| *Tributary* | | | | | | | | | | | | | | | |  |  |
| U1 | 111.5296 | 21.71809 | |  | | 0.20 | | 40 | 0.21 | |  | | 23.0 | 0.38 | 0.88 | |  |
| U2 | 111.5411 | 21.69545 | |  | | 0.10 | | 26 | 0.47 | |  | | 7.90 | 0.31 | 0.54 | |  |
| U3 | 111.5699 | 21.69473 | |  | | 0.20 | | 18 | 0.16 | |  | | 14.5 | 0.18 | 0.52 | |  |
| U4 | 111.6019 | 21.71642 | |  | | 0.40 | | 27 | 0.68 | |  | | 11.3 | 0.17 | 0.84 | |  |
| U5 | 111.6062 | 21.73660 | |  | | 0.10 | | 20 | 0.24 | |  | | 14.7 | 0.28 | 0.96 | |  |
| M1 | 111.6276 | 21.73911 | |  | | 0.30 | | 32 | 0.23 | |  | | 36.3 | 0.98 | 1.4 | |  |
| M2 | 111.6446 | 21.74415 | |  | | 0.30 | | 17 | 1.03 | |  | | 16.7 | 0.26 | 0.78 | |  |
| L1 | 111.6764 | 21.77746 | |  | | 0.10 | | 16 | 1.58 | |  | | 41.6 | 0.34 | 0.56 | |  |
| L2 | 111.6831 | 21.78608 | |  | | 0.020 | | 15 | 0.55 | |  | | 40.6 | 0.98 | 1.5 | |  |

**Table S****2.** The normal pollutants indexes of water and sediments sampled from Zhilong river basin in Yangjiang city of Guangdong, China.

| Index | Water (mg L^-1^) | | | Sediment (g kg^-1^) | | |
| --- | --- | --- | --- | --- | --- | --- |
|  | TP | COD | NH_3_-N | TOC | TN | TP |
| Mainstream | | | | | | |
| min | 0.00 | 14 | 0.14 | 2.96 | 0.10 | 0.17 |
| max | 0.16 | 25 | 0.58 | 11.5 | 0.27 | 0.50 |
| average | 0.080 | 19 | 0.36 | 6.30 | 0.21 | 0.32 |
| Standard deviation | 0.070 | 5.0 | 0.19 | 3.87 | 0.080 | 0.15 |
| Variable coefficient (%) | 90.1 | 26.1 | 52.2 | 61.5 | 37.4 | 47.2 |
| Tributary | | | | | | |
| min | 0.020 | 15 | 0.16 | 7.90 | 0.17 | 0.52 |
| max | 0.41 | 40 | 1.6 | 41.6 | 0.98 | 1.5 |
| average | 0.20 | 23 | 0.57 | 23.0 | 0.43 | 0.88 |
| Standard deviation | 0.12 | 8.5 | 0.47 | 13.1 | 0.32 | 0.35 |
| Variable coefficient (%) | 61.2 | 36.2 | 82.6 | 57.2 | 73.5 | 39.6 |

**Table S3.** The sediment quality and potential ecological risk assessment method Levels of the geo-accumulation index (*I_geo_*) in relation to sediment quality.

| Levels of assessment method | Contamination levels |
| --- | --- |
| The geo-accumulation index (*I_geo_*) | |
| I_geo_ | Sediment quality |
| I_geo_ ≤ 0 | Practically uncontaminated |
| 0 < I_geo_ ≤ 1 | Uncontaminated to moderately contaminated |
| 1 < I_geo_ ≤ 2 | Moderately contaminated |
| 2 < I_geo_ ≤ 3 | Moderately to heavily contaminated |
| 3 < I_geo_ ≤ 4 | Heavily contaminated |
| 4 < I_geo_ ≤ 5 | Heavily to extremely contaminated |
| I_geo_ > 5 | Extremely contaminated |
| Risk assessment coding (RAC) method | |
| RAC | Ecological risk levels |
| RAC < 1 | No risk |
| 1 ≤ RAC < 10 | Low risk |
| 10 ≤ RAC < 30 | Moderate risk |
| 30 ≤ RAC < 50 | High risk |
| RAC ≥ 50 | Very high risk |
| Sediment quality guideline | |
| TEL | Threshold effect levels |
| C<TEL | No impact |
| TEL >C<PEL | Occasionally toxic |
| C>PEL | Frequently toxic |

**Table S4.** Background concentrations (BC), Threshold effect levels (TEL) and probable effect levels (PEL) of heavy metals (mg kg^-1^) used in this study.

| Metal(loid)s | BC ^a^ | TEL | PEL |
| --- | --- | --- | --- |
| Cd | 0.11 | 0.596 | 3.539 |
| Pd | 60 | 35 | 91.3 |
| Cu | 32 | 35.7 | 197 |
| Ni | 28 | 18 | 36 |
| Cr | 77 | 37.3 | 90 |

^a^ The values was the soil background concentrations of Guangdong Province.

**Table S5.** The results of heavy metals in the sediments analyzed by geo-accumulation index (*I_geo_*), risk assessment coding (RAC), and Sediment quality guideline methods

| Metal(loid)s | Geo-accumulation index (I_geo_) ^a^ | | | | | Risk assessment coding (RAC) ^b^ | | | | | Sediment quality guideline ^c^ | | | | |
| --- | --- | --- | --- | --- | --- | --- | --- | --- | --- | --- | --- | --- | --- | --- | --- |
|  | Cd | Cd | Pb | Cu | Ni | Cd | Pb | Cu | Ni | Cr | Cd | Pb | Cu | Ni | Cr |
| S1 | a | a | a | a | a | 4 | 1 | 3 | 1 | 3 | A | A | A | A | A |
| S2 | a | a | a | a | a | 1 | 1 | 2 | 1 | 2 | A | A | A | B | B |
| S3 | a | a | a | a | a | 1 | 1 | 1 | 1 | 4 | A | B | A | A | A |
| S4 | a | a | a | a | a | 4 | 1 | 3 | 1 | 2 | A | A | A | A | A |
| U1 | a | a | a | a | a | 1 | 1 | 3 | 1 | 2 | A | A | A | A | A |
| U2 | a | a | a | a | a | 1 | 1 | 4 | 1 | 2 | A | B | A | A | A |
| U3 | a | a | a | a | a | 3 | 1 | 2 | 2 | 2 | A | B | A | B | B |
| U4 | b | a | a | a | a | 5 | 1 | 2 | 2 | 2 | A | B | A | B | A |
| U5 | a | a | a | a | a | 1 | 1 | 4 | 1 | 2 | A | A | A | A | A |
| M1 | a | a | a | a | a | 4 | 1 | 3 | 1 | 2 | A | B | A | A | A |
| M2 | a | a | a | a | a | 5 | 1 | 3 | 1 | 2 | A | A | A | A | A |
| L1 | a | a | a | a | a | 5 | 1 | 2 | 1 | 2 | A | A | A | A | A |
| L2 | a | a | a | a | a | 3 | 1 | 3 | 1 | 2 | A | A | A | A | A |

^a^ “a” and “b” represented “practically uncontaminated” and “incontaminated to moderately contaminated”, respectively”;

^b^ “1”, “2”, “3”, “4”, “5” represented “No risk”, “Low risk”, “Moderate risk”, “High risk” and “Very high risk”;

^c^ “A”, “B”, and “C” represented “No impact”, “Moderate risk” and “Occasionally toxic”.

**Table S6.** The relationships of genus abundance in sediments between different sampling sites.

| Sampling sties | S1 | S2 | S3 | S4 |
| --- | --- | --- | --- | --- |
| S1 | 1 | .245 | -.017 | .238 |
| S2 | .245 | 1 | .555^**^ | .946^**^ |
| S3 | -.017 | .555^**^ | 1 | .470^**^ |
| S4 | .238 | .946^**^ | .470^**^ | 1 |
| U1 | .250 | .878^**^ | .393^*^ | .963^**^ |
| U2 | .362^*^ | .837^**^ | .409^*^ | .806^**^ |
| U3 | .256 | .958^**^ | .437^*^ | .976^**^ |
| U4 | .088 | -.074 | .001 | -.092 |
| U5 | -.312 | -.166 | -.257 | -.162 |
| M1 | .232 | .992^**^ | .541^**^ | .954^**^ |
| M2 | .228 | .968^**^ | .474^**^ | .969^**^ |
| L1 | .204 | .345 | .291 | .333 |
| L2 | .021 | .192 | .281 | .039 |

**Table S7.** The relationships between different MRGs (top 20 for abundance) in sediments sampled from Zhilong River basin in Yangjiang city of Guangdong, China.

|  | zraR/hydH | modC | corR | ruvB | fbpC | copR | pstB | arsT | znuC/yebM | wtpC | acn | copA | nrsS | arsM | arsB | nikE | tupC | nikD | baeR | arsC |
| --- | --- | --- | --- | --- | --- | --- | --- | --- | --- | --- | --- | --- | --- | --- | --- | --- | --- | --- | --- | --- |
| zraR/hydH | 1.000 |  |  |  |  |  |  |  |  |  |  |  |  |  |  |  |  |  |  |  |
| modC | .225 | 1.000 |  |  |  |  |  |  |  |  |  |  |  |  |  |  |  |  |  |  |
| corR | .571^*^ | .835^**^ | 1.000 |  |  |  |  |  |  |  |  |  |  |  |  |  |  |  |  |  |
| ruvB | .104 | .110 | .214 | 1.000 |  |  |  |  |  |  |  |  |  |  |  |  |  |  |  |  |
| fbpC | .214 | .846^**^ | .725^**^ | .088 | 1.000 |  |  |  |  |  |  |  |  |  |  |  |  |  |  |  |
| copR | .549 | .505 | .478 | -.044 | .505 | 1.000 |  |  |  |  |  |  |  |  |  |  |  |  |  |  |
| pstB | -.060 | -.780^**^ | -.747^**^ | -.203 | -.566^*^ | -.104 | 1.000 |  |  |  |  |  |  |  |  |  |  |  |  |  |
| arsT | .390 | .302 | .390 | .346 | .044 | .544 | -.297 | 1.000 |  |  |  |  |  |  |  |  |  |  |  |  |
| znuC/yebM | .374 | .533 | .478 | .005 | .582^*^ | .857^**^ | -.203 | .500 | 1.000 |  |  |  |  |  |  |  |  |  |  |  |
| wtpC | .154 | 0.000 | .011 | .269 | .253 | -.022 | .379 | -.379 | .049 | 1.000 |  |  |  |  |  |  |  |  |  |  |
| acn | .429 | .615^*^ | .714^**^ | .473 | .280 | .478 | -.681^*^ | .769^**^ | .374 | -.357 | 1.000 |  |  |  |  |  |  |  |  |  |
| copA | -.198 | -.912^**^ | -.841^**^ | -.280 | -.698^**^ | -.412 | .912^**^ | -.495 | -.511 | .247 | -.786^**^ | 1.000 |  |  |  |  |  |  |  |  |
| nrsS | .165 | -.654^*^ | -.538 | -.126 | -.418 | .011 | .923^**^ | -.148 | -.170 | .418 | -.555^*^ | .808^**^ | 1.000 |  |  |  |  |  |  |  |
| arsM | .599^*^ | -.154 | -.022 | .143 | -.137 | .137 | .407 | .154 | -.033 | .533 | -.038 | .302 | .549 | 1.000 |  |  |  |  |  |  |
| arsB | .077 | -.654^*^ | -.599^*^ | -.066 | -.478 | -.192 | .857^**^ | -.176 | -.280 | .467 | -.604^*^ | .780^**^ | .912^**^ | .632^*^ | 1.000 |  |  |  |  |  |
| nikE | .247 | .879^**^ | .786^**^ | .275 | .654^*^ | .407 | -.775^**^ | .467 | .549 | .038 | .681^*^ | -.890^**^ | -.676^*^ | -.033 | -.571^*^ | 1.000 |  |  |  |  |
| tupC | .126 | -.632^*^ | -.555^*^ | -.071 | -.423 | -.027 | .874^**^ | -.077 | -.099 | .505 | -.544 | .775^**^ | .912^**^ | .665^*^ | .890^**^ | -.511 | 1.000 |  |  |  |
| nikD | -.077 | .643^*^ | .522 | .143 | .478 | .192 | -.830^**^ | .242 | .170 | -.368 | .604^*^ | -.720^**^ | -.830^**^ | -.335 | -.835^**^ | .610^*^ | -.731^**^ | 1.000 |  |  |
| baeR | .110 | -.566^*^ | -.533 | -.242 | -.368 | .143 | .874^**^ | -.170 | -.060 | .429 | -.500 | .775^**^ | .852^**^ | .571^*^ | .714^**^ | -.577^*^ | .879^**^ | -.538 | 1.000 |  |
| arsC | .319 | -.341 | -.286 | .352 | -.143 | .407 | .599^*^ | .236 | .264 | .401 | -.066 | .396 | .665^*^ | .544 | .571^*^ | -.231 | .709^**^ | -.390 | .665^*^ | 1.000 |

**Table S8.** Mean concentrations (mg kg^-1^) of metal(loid)s in the sediments of this study and other urban rivers of China.

| Rivers | Pb | Ni | Cr | Cu | Cd |
| --- | --- | --- | --- | --- | --- |
| Zhilong River (this study) | 33.48 | 10.65 | 22.23 | 9.88 | 0.06 |
| Ganjiang River^a^ | 22.33 | n.a. | 20.20 | 29.51 | 2.34 |
| Lower Lijiang River^b^ | 40.25 | 20.36 | 39.52 | 21.90 | 0.87 |
| Maozhou River^c^ | 60.26 | 370.06 | 609.59 | 1212.80 | 1.17 |
| Lower Yangtze River^d^ | 41.90 | n.a. | 84.93 | 46.58 | 0.66 |
| Daqing River^e^ | 32.01 | 34.74 | 110.28 | 73.91 | 0.68 |
| Xiaoqing River^f^ | 48.42 | 34.13 | 177.87 | 46.81 | 2.38 |
| Huangshui River^g^ | 43.49 | 46.19 | 89.63 | 23.07 | 0.25 |
| Middle reach of the Yarlung Zangbo River^h^ | 23.12 | 79.59 | 135.34 | 39.9 | 0.08 |

^a^ (Shi et al., 2019); ^b^ (Xiao et al., 2021); ^c^ (Gong et al., 2016); ^d^ (Wang et al., 2018); ^e^ (Tang et al., 2015); ^f^ (Jiao et al., 2017); ^g^ (Bai et al., 2014).

**Table S9.** The Microbial diversity index in sediments sampled from Zhilong River.

| Sampling sites | Chao1 | Observed_features | Shannon_entropy | Simpson |
| --- | --- | --- | --- | --- |
| S1 | 1415.016 | 1325 | 5.109403 | 0.820622 |
| S2 | 2331.226 | 2211 | 5.326904 | 0.842816 |
| S3 | 2555.938 | 2404 | 5.95617 | 0.891782 |
| S4 | 2375.254 | 2228 | 5.752368 | 0.893805 |
| U1 | 1607.561 | 1522 | 5.165264 | 0.845023 |
| U2 | 1786.923 | 1680 | 5.125439 | 0.848807 |
| U3 | 1932.422 | 1820 | 5.081556 | 0.848353 |
| U4 | 1560.784 | 1481 | 5.095663 | 0.861856 |
| U5 | 2224.952 | 2124 | 5.441468 | 0.937032 |
| M1 | 2145.178 | 2022 | 5.212385 | 0.842249 |
| M2 | 1873.429 | 1722 | 4.975866 | 0.833857 |
| L1 | 1095.322 | 1044 | 3.952102 | 0.771104 |
| L2 | 2439.930 | 2285 | 6.293485 | 0.937024 |

**
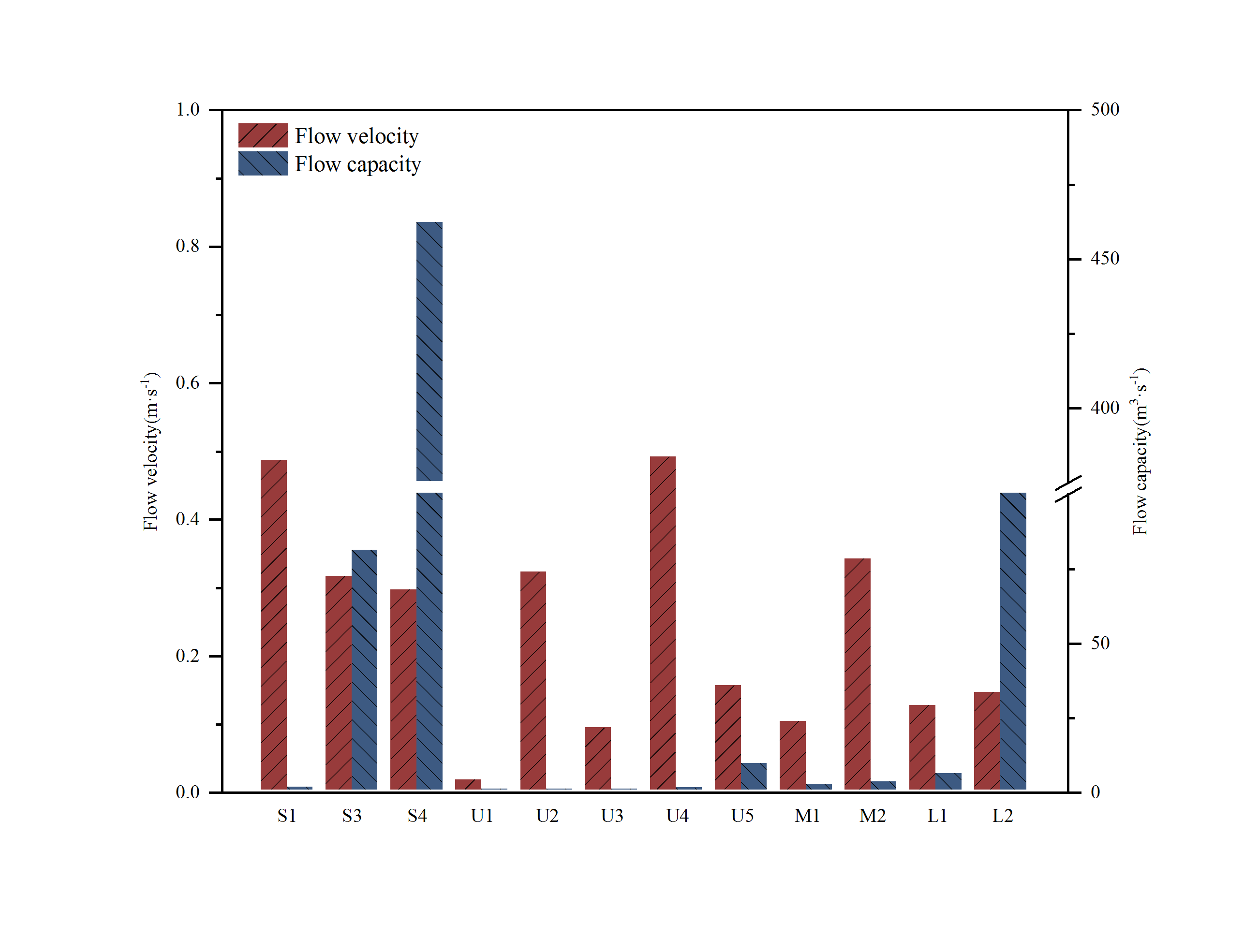
Figure S1.** The flow velocity and flow rate of mainstream and tributaries in Zhilong River Basin.


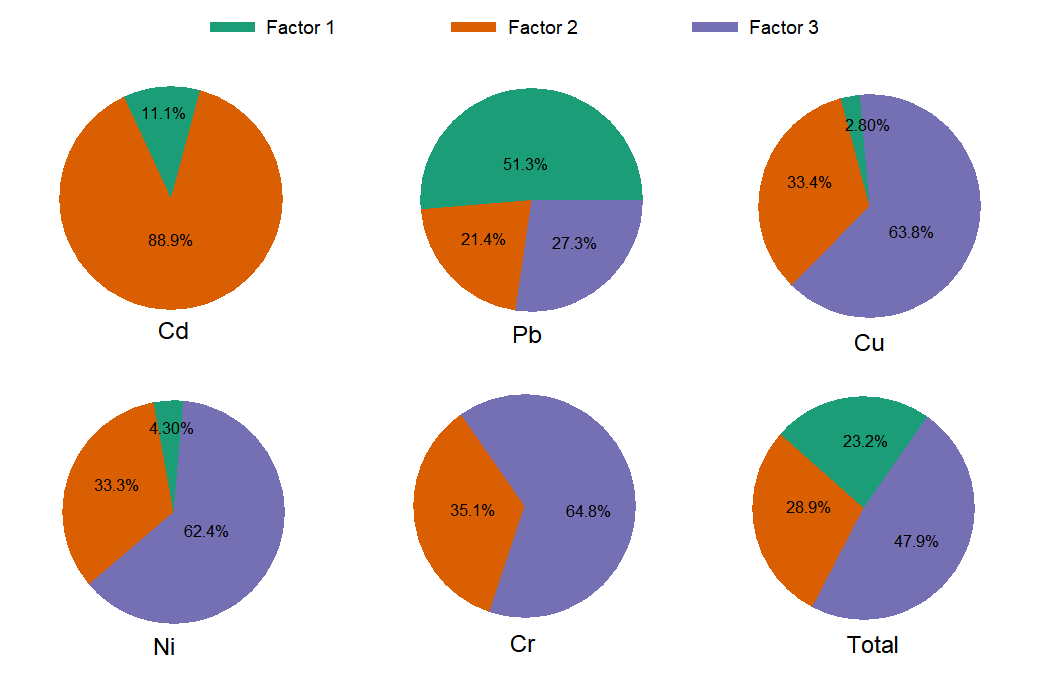
**Figure S2.** Source apportionment of sedimentary heavy metals based on positive matrix factorization (PMF) model.


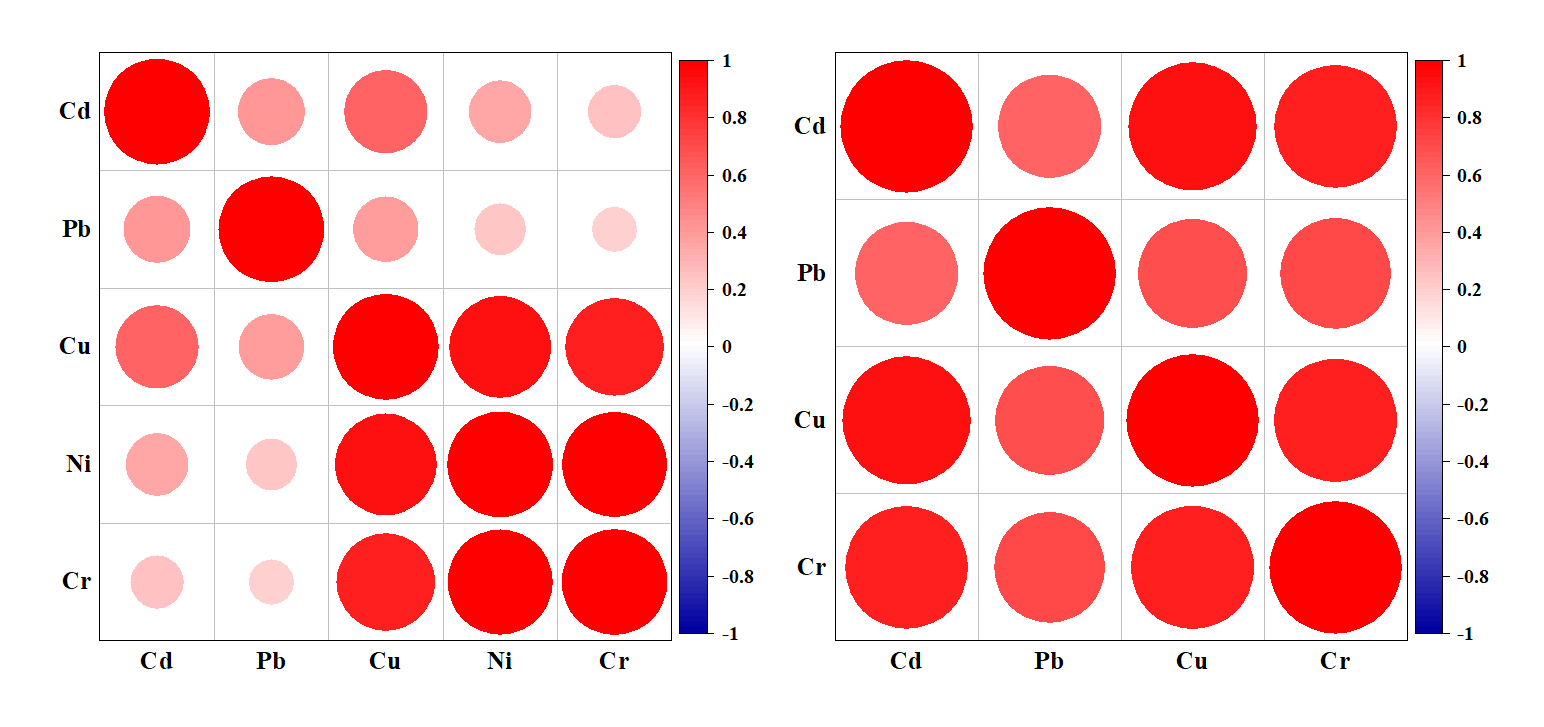


**Figure S3.** The relationships between different (a) total and (b) mobile fractions concentrations of heavy metal in sediments sampled from Zhilong River basin in Yangjiang city of Guangdong, China.

**Figure S4.** The concentrations of TP and COD in water sampled from the mainstream and tributaries of Zhilong River Basin in Yangjiang of Guangdong, China.


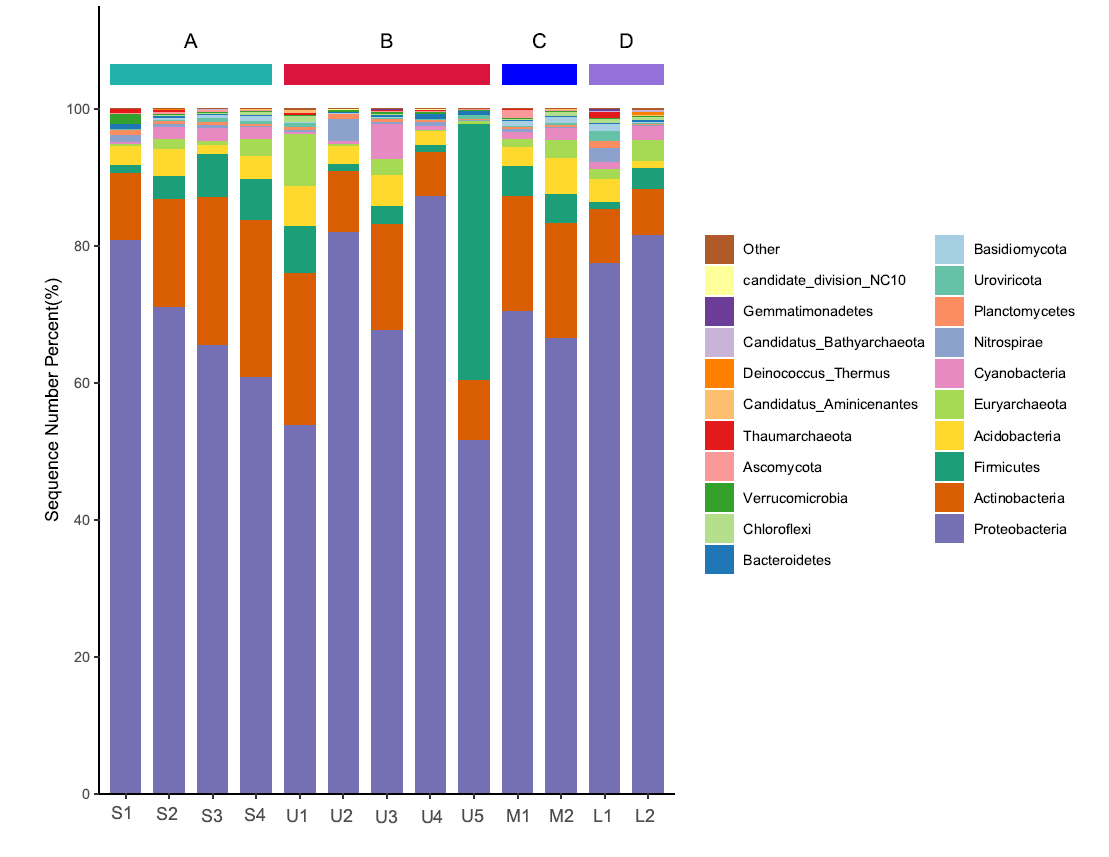


**Figure S5.** Taxonomic composition of microbial communities of Zhilong River at the phylum level among different river sediment. Different colors refer to different phyla.


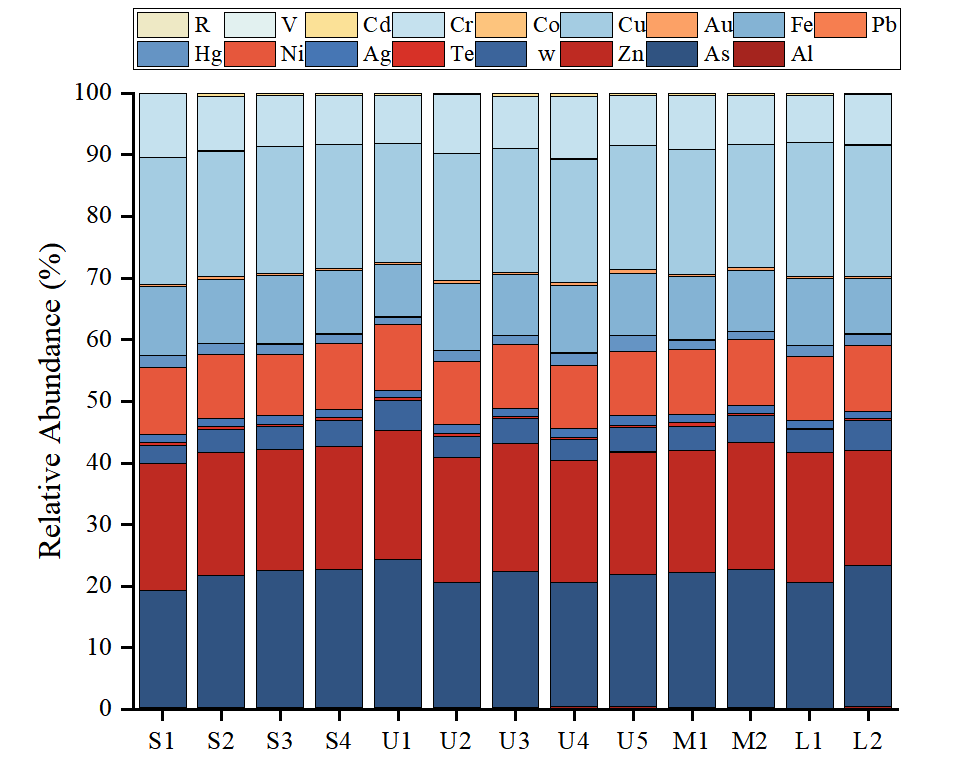


**Figure S6.** Relative abundance of heavy metals in sediments from the Zhilong River.


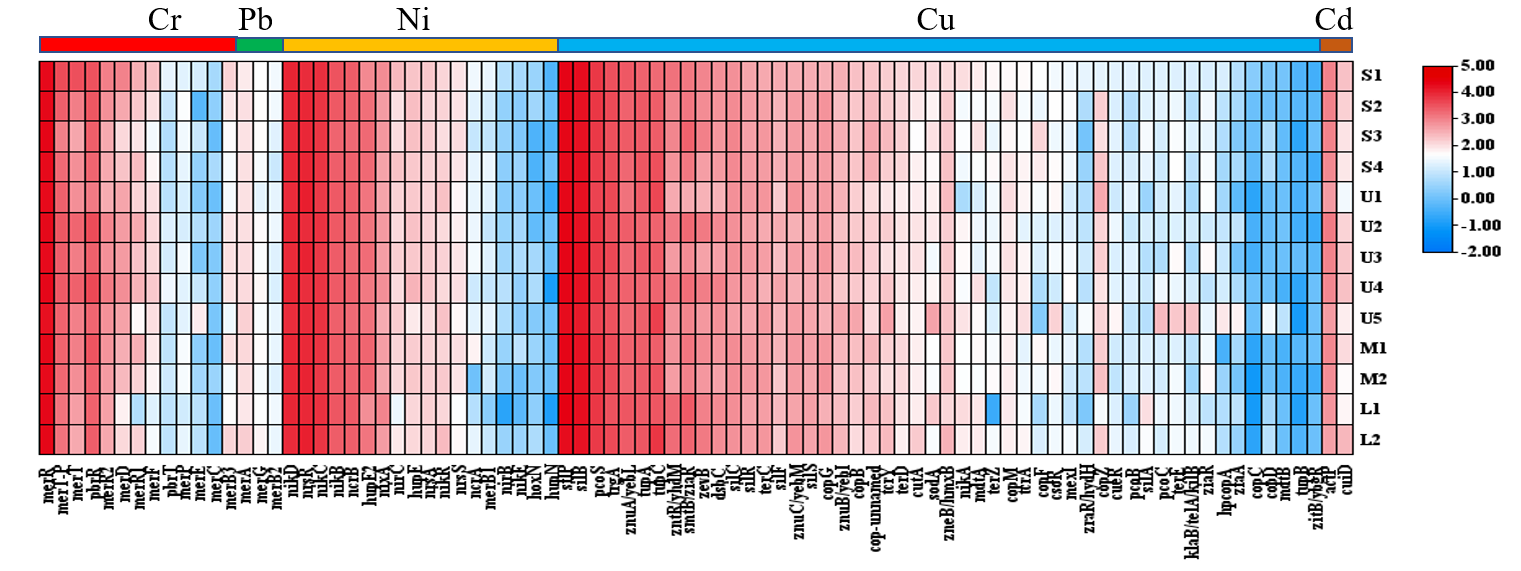


**Figure S7.** Heatmap of MRG levels for sediment in mainstream and tributary of Zhilong River basin.

**
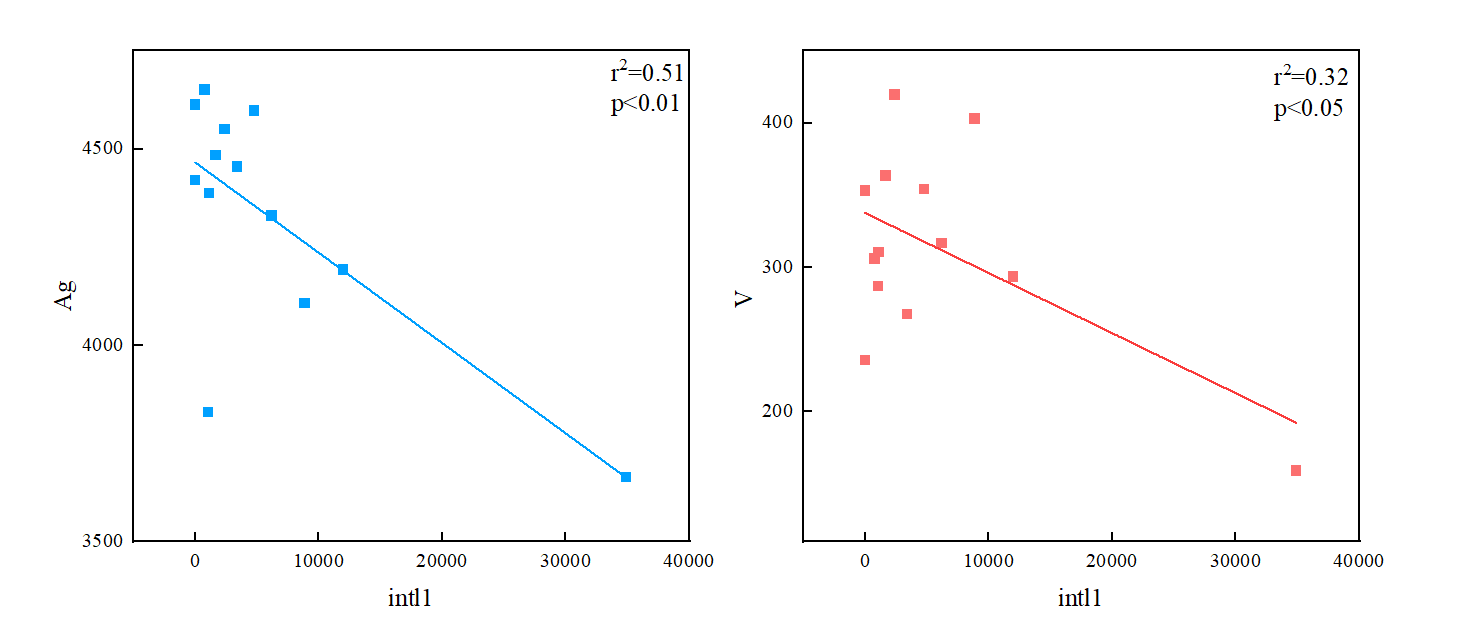
Figure S8.** The abundance relationships between *intl1* and metal elements (Ag and V) in sediments sampled from Zhilong River basin.

**Reference**

Bai J.K., Li C.L., Kang S.C., Chen P.F., Wang J.L., (2014). Chemical speciation and risk assessment of heavy metals in the middle part of Yarlung Zangbo surface sediments. *Huanjing Kexue (in Chinese)* 35, 3346-3351.

Gong Y.L., Huang C., Huang L., Li H.Y., Lin X.Y., Xu C., Tan L.M., Zhao L., (2016). Ecological risk assessment of heavy metals in the surface sediments of Maozhou River. *Jishou Daxue Xuebao (in Chinese)* 37, 35-39.

Jiao F., Ren L., Wang X., Liu W., (2017). Pollution characteristics and potential ecological risk assessment of metals in the sediments of Xiaoqing River, Jinan. *Environ Sci Pollut Res Int* 24, 15001-15011. doi.org/10.1007/s11356-017-9056-8.

Shi X., Zhang W., (2019). Heavy metal pollution and spatial distribution in Surface Water and Sediment of Nanchang Section of Ganjiang River. *IOP Conference Series: Earth and Environmental Science* 300. doi.org/10.1088/1755-1315/300/3/032045.

Tang W.Z., Wang L.S., Shan B.Q., Zhao Y., Su H.C., L. Y., (2015). Heavy metal pollution of the surface sediments in Daqing River System, Haihe Basin. *Acta Scientiae Circumstantiae (in Chinese)* 35, 3620-3627.

Wang H., Wu Q., Hu W., Huang B., Dong L., Liu G., (2018). Using multi-medium factors analysis to assess heavy metal health risks along the Yangtze River in Nanjing, Southeast China. *Environ Pollut* 243, 1047-1056. doi.org/10.1016/j.envpol.2018.09.036.

Xiao H., Shahab A., Xi B., Chang Q., You S., Li J., Sun X., Huang H., Li X., (2021). Heavy metal pollution, ecological risk, spatial distribution, and source identification in sediments of the Lijiang River, China. *Environ Pollut* 269, 116189. doi.org/10.1016/j.envpol.2020.116189.
